# Rapid and specific on-site H5Nx avian influenza diagnosis via RPA and PAM-independent CRISPR-Cas12a assay combined with anti-NP antibody-based viral RNA purification

**DOI:** 10.3389/fvets.2025.1520349

**Published:** 2025-01-17

**Authors:** Jin-Ha Song, Seung-Eun Son, Ho-Won Kim, Seung-Ji Kim, Se-Hee An, Chung-Young Lee, Hyuk-Joon Kwon, Kang-Seuk Choi

**Affiliations:** ^1^Laboratory of Avian Diseases, College of Veterinary Medicine and BK21 PLUS for Veterinary Science, Seoul National University, Seoul, Republic of Korea; ^2^Avian Influenza Research and Diagnostic Division, Animal and Plant Quarantine Agency, Gimcheon-si, Republic of Korea; ^3^Department of Microbiology, College of Medicine, Kyungpook National University, Daegu, Republic of Korea; ^4^Research Institute for Veterinary Science, College of Veterinary Medicine, Seoul National University, Seoul, Republic of Korea; ^5^Laboratory of Poultry Medicine, Department of Farm Animal Medicine, College of Veterinary Medicine and BK21 PLUS for Veterinary Science, Seoul National University, Seoul, Republic of Korea; ^6^Farm Animal Clinical Training and Research Center (FACTRC), GBST, Seoul National University, Pyeongchang, Republic of Korea; ^7^GeNiner Inc., Seoul, Republic of Korea

**Keywords:** avian influenza virus, magnetic beads, ribonucleoprotein purification, CRISPR-Cas12a, PAM-independent, on-site detection

## Abstract

Rapid and accurate detection of H5Nx avian influenza viruses is critical for effective surveillance and control measures. Currently, RT-qPCR with spin column RNA extraction is the gold standard for HPAIV surveillance, but its long reaction time and need for specialized equipment limit its effectiveness for rapid response. In this study, we introduce a centrifuge-free, rapid detection method for on-site detection of H5Nx viruses that combines magnetic bead-based ribonucleoprotein (RNP) purification and concentration with a CRISPR-Cas12a system that is independent of the protospacer adjacent motif (PAM) sequence. Our approach employs anti-NP monoclonal antibodies for the targeted isolation of RNA bound to RNPs, facilitating a quick and specific RNA extraction process that negates the need for centrifugation. Additionally, by denaturing the RT-RPA amplicon using 60% DMSO, we activate the trans-ssDNA cleavage activity of the Cas12a protein without the need for a specific PAM (5’-TTTV-3′) sequence. This strategy increases flexibility in CRISPR RNA design, providing a significant advantage when targeting genes with high variability. We validated the efficacy of our magnetic RNP purification and concentration method in combined with an RT-RPA/PAM-independent Cas12a assay for detecting the H5 gene. The assay achieved a sensitivity threshold of 10^1^ EID_50_ with fluorescent detection and 10^2^ EID_50_ using lateral flow strips. It also exhibited high specificity, yielding positive results solely for H5Nx viruses among various influenza A virus subtypes. Furthermore, in clinical samples, the assay demonstrated 80% sensitivity and 100% specificity. These results highlight the advantages of using NP-specific antibodies for RNP purification and CRISPR-Cas12a with viral gene-specific crRNA to achieve exceptional diagnostic specificity.

## 1 Introduction

Avian influenza viruses (AIVs) belong to the *Orthomyxoviridae* family and are characterized by their 8 RNA genome segments and a variety of structural and nonstructural proteins. AIVs from wild waterfowl are classified on the basis of the antigenic structures of 16 HA (H1-H16) and 9 NA (N1-N9) surface proteins. Highly pathogenic avian influenza virus (HPAIV) subtypes, such as H5Nx or H7Nx, are distinguished by the presence of multiple basic amino acids at the cleavage site (1). Among HPAIVs, the H5 subtype has been responsible for significant global outbreaks in wild birds and poultry across Asia, Europe, Africa, and North America since 2014 (2). Despite regular annual surveillance in Korea, complete prevention of H5Nx HPAIV remains challenging due to the high transmissibility and mutation rate of the virus and its spread through migratory birds. As a result, many affected countries face difficulties in preventing the virus from entering poultry farms. Since 2016, clade 2.3.4.4 subgroup B H5Nx viruses have been introduced into Korea, causing significant damage to the domestic poultry industry (3–5).

To mitigate the impact of HPAIVs, it is imperative to implement rapid and accurate diagnostic measures during the early stages of viral outbreaks. Currently, the combination of RNA extraction and quantitative reverse transcription polymerase chain reaction (RT–qPCR) is the gold-standard method used for HPAIV surveillance. However, the lengthy reaction time and requirement for specialized equipment can make this a cumbersome method, making it challenging to respond rapidly to HPAIV occurrences. On the other hand, immunochromatography is a relatively simple and rapid detection method but lacks the necessary sensitivity for field application (6).

Recently, various molecular diagnostic methods have emerged, including isothermal amplification techniques, nanoparticle-based diagnostics, and clustered regularly interspaced short palindromic repeats (CRISPR)-Cas system diagnostics, which can be applied in the field without the need for specialized expertise or equipment (7–9). Recombinase polymerase amplification (RPA) is highly sensitive and selective isothermal amplification technique that can be performed at temperatures between 37 and 42°C and produces results within 20 min (10, 11). Although RPA offers rapid reactions, it usually yields nonspecific amplification signals (12). To address this, CRISPR, a third-generation gene editing technology, is employed as an amplification product detection method to reduce nonspecific reactions during isothermal amplification. This has garnered attention as a next-generation disease diagnosis technology (9, 13–16). Most diagnostic methods utilizing CRISPR/Cas technology employ either the Cas13 (9, 17, 18) or Cas12 (13–15) enzyme, both of which have trans-cleavage activity. While Cas13 does not require a protospacer-adjacent motif (PAM) sequence (5’-TTTV-3′), its spacer length of 28–30 nucleotides is relatively long compared with the 20-nucleotide spacer of Cas12, making CRISPR RNA (crRNA) design more challenging for the highly variable H5 gene. Additionally, because Cas13 targets RNA, an extra *in vitro* transcription step is necessary for postgene amplification. In contrast, Cas12 targets DNA, eliminating the need for in vitro transcription, and its shorter 20-nucleotide spacer length simplifies crRNA design for conserved regions within highly variable sequences. However, for the Cas12 enzyme to be activated, it must recognize a T nucleotide-rich PAM sequence at the 5′ end of the target dsDNA (5’-TTTV-3′) (13). This requirement complicates crRNA design for the highly variable H5 gene.

To overcome this challenge, we developed a CRISPR/Cas12a assay that does not require consideration of the PAM sequence, facilitating the design of crRNA for the hypervariable H5 gene. Additionally, we developed a method to purify and concentrate RNA via magnetic beads and virus-specific antibodies. Given that RNA is bound to the surface of ribonucleoproteins (RNPs) (19), we developed RNP purification and concentration techniques using virus-specific antibody-magnetic bead complex. By combining the magnetic bead-based RNP purification and concentration method with a PAM-independent CRISPR/Cas12a detection system, we developed a technique that exhibited remarkable promise in the rapid, highly sensitive, and specific detection of H5Nx viruses.

## 2 Materials and methods

### 2.1 Viruses and clinical samples

All the viruses used in this study to measure diagnostic sensitivity and specificity are listed in Table 1 and were propagated in 10-day-old SPF embryonated chicken eggs (ECEs) (VALO, Biomedia GmbH). The titer of each virus was determined as the 50% egg infectious dose (EID_50_) by serially inoculating 10-fold diluted virus into 5 10-day-old SPF eggs. After the virus-inoculated eggs were incubated at 37°C for 72 h, the EID_50_ was determined via the hemagglutination test for each dilution, with calculations performed by the Spearman–Karber method (20). The clinical samples used in this study were fecal samples collected from migratory bird habitats in South Korea. All fecal samples were suspended in PBS at a 1:10 ratio and consisted of 15 RT-qPCR-positive samples and 14 RT-qPCR-negative samples. All RT-qPCR-positive samples were verified through virus isolation in ECEs.

**Table 1:** List of viruses used for the specificity test and the number of mismatches with the primer/crRNA set.

Strain name	Subtype	Abbreviation	Forward primer mismatch	Reverse primer mismatch	crRNA mismatch	Total mismatch
A/*Anser fabalis*/South Korea/180371/2018	H1N5	180,371	12	5	1	18
A/*Anas platyrhynchos*/South Korea/1811160/2018	H7N5	1,811,160	12	6	7	25
A/*Anser fabalis*/South Korea/19NV-50/2019	H6N2	19NV-50	7	3	2	12
A/*Anser fabalis*/South Korea/19 DC-15/2019	H6N2	19 DC-15	7	4	2	13
A/*Anser fabalis*/South Korea/19 DC-20/2019	H6N2	19 DC-20	7	4	2	13
A/*Anser brachyrhynchus*/South Korea/19 DC-42/2019	H6N1	19 DC-42	7	4	2	13
A/*Anser brachyrhynchus*/South Korea/19 DC-44/2019	H11N2	19 DC-44	7	8	5	20
A/*Anser albifrons*/South Korea/22JN-163/2022	H10N7	22JN-163	11	8	7	26
A/*Anser albifrons*/South Korea/22MC-41/2022	H6N2	22MC-41	7	4	3	14
A/chicken/Korea/SL20/2020	H9N2	SL20	9	7	4	20
A/Greylag Goose/South Korea/SW21/2021	H9N2	SW21	6	6	3	15
A/Mandarin duck/Korea/K10-483/2010,2.3.2.1 c rH5N1(p) *	H5N1	K10-483	0	1	0	1
2.3.4.4a rH5N8(p)*^a^	H5N8	rH5N8	0	0	0	0
2.3.4.4c rH5N6(p)*^b^	H5N6	rH5N6	1	1	1	3
2.3.4.4b rH5N1(p)*^c^	H5N1	rH5N1	2	2	0	4
A/wild duck/korea/SNU50-5/2009	H5N1	SNU50-5	0	0	0	0
A/chicken/Korea/01310/2001	H9N2	01310	6	10	3	19
A/Korea/KBNP-0028/2000	H9N2	0028	7	10	3	20
A/Puerto Rico/8/34	H1N1	PR8	10	9	3	22
A/canine/Korea/SH6/2017 (CIV)	H3N2	CIV	10	5	7	22
Korea/SNU19018/19, Infectious bronchitis (IB),		IBV				

*The 2 + 6 recombinant viruses generated through reverse genetics. The HA and NA gene segments were derived from those listed in the table, while the remaining six internal gene segments were derived from the PR8 strain.

a Recombinant H5N8 influenza virus generated using consensus HA and NA sequences from clade 2.3.4.4a H5N8 HPAIVs isolated in Asia between 2014 and 2016, with the multi-basic cleavage site in the HA gene replaced by an ASGR-coding sequence to attenutae virulence.

b Recombinant H5N6 influenza virus generated using most frequent HA and NA sequences from clade 2.3.4.4c H5N6 HPAIVs isolated between 2014 and 2016, with the multi-basic cleavage site in the HA gene replaced by an ASGR-coding sequence to attenutae virulence.

c Recombinant H5N1 influenza virus generated using consensus HA and NA sequences from clade 2.3.4.4b H5N1 HPAIVs isolated in Asia between 2021 and 2023, with the multi-basic cleavage site in the HA gene replaced by an ASGR-coding sequence to attenutae virulence.

### 2.2 Bioinformatics

A total of 8,504 H5 gene sequences of H5Nx IAVs isolated between 1977 and 2020 were obtained from the Global Initiative on Sharing All Influenza Data (GISAID).^1^ These sequences were subjected to multiple-sequence alignment via the MAFFT online tool (21). The resulting consensus sequence was analyzed, and a highly conserved 200 bp region was selected. To compare nucleotide variability within this selected region, positional nucleotide numerical summary (PNNS) and entropy calculations were conducted via a website,^2^ following previously published methods (22). To analyze the number and frequency of mismatches between the selected forward primer/reverse primer/crRNA and the 8,504 H5 gene sequences used in the study, we cropped the sequences at the corresponding positions of the HA gene for each isolate. We then analyzed the number of mismatched sequences and the frequency of these viruses (Supplementary data). To analyze the mismatches between the selected primers/crRNAs and the recently isolated clade 2.3.2.1c or 2.3.4.4b HPAIV H5Nx strains (from 2020 to 2024), we downloaded the H5 gene sequences and compared them with the selected primers/crRNAs via the method described above.

### 2.3 Conventional RNA extraction, RT-RPA and PAM-independent Cas12a detection

Viral RNA was extracted via a conventional spin–column kit (RNA Gene-spin, iNtRON Biotechnology) following the manufacturer’s protocol. For the RT-RPA assay, we utilized a TwistAmp Basic Kit (TwistDx) according to the manufacturer’s protocol with slight modifications. We resuspended the lyophilized RPA pellet in a solution containing 29.5 μL of rehydration buffer, 1 μL of 20 mM forward and reverse primers, 6.5 μL of RNase-free water, 1 μL of reverse transcriptase (Thermo Scientific), and 0.5 μL of RNase Inhibitor (Enzynomics). We subsequently aliquoted 7 μL of this mixture into a 0.2 mL PCR tube, added 2.5 μL of extracted RNA or purified vRNP, and finally added 0.5 μL of MgOAc, followed by a 20-min reaction at 42°C. After the 20-min reaction period, we added 15 μL of pure dimethylsulfoxide (DMSO) (Duchefa Biochemie) to the reaction mixture. We subsequently introduced a premixed crRNA-Cas12a complex consisting of 0.5 μL of 1 μM crRNA (Integrated DNA Technologies, IDT) and 0.5 μL of 1 μM EnGen Lba Cas12a (NEB), along with 1 μL of 10x NEBuffer 2.1 and 2 μL of 10 μM ssDNA FQ probe reporter (Bionics). This mixture was then subjected to another 20-min reaction at 42°C. The fluorescence kinetics were measured using a Bio-Rad CFX Connect real-time PCR detection system. For the lateral flow readout, all procedures were identical to those for the fluorescence detection method, except that we used a reporter labeled with biotin at the 3′ end. After the final 20-min reaction at 42°C, we inserted a lateral flow strip (LFS; Milenia HybriDetect, TwistDx) into the tube and incubated at RT for 3 min. To optimize the PAM-independent Cas12a detection system, RT-RPA amplicons were treated with various concentrations of DMSO (0, 20, 40, 60, and 80%), followed by the PAM-independent Cas12a assay, and fluorescence kinetics were measured as described above. Additionally, the optimal reaction temperature for both the RT-RPA and PAM-independent Cas12a detection assays was determined by performing the assays at six different temperatures (20°C, 30°C, 37°C, 42°C, 46°C, and 50°C) and analyzing the fluorescence kinetics.

### 2.4 Preparation of NP monoclonal antibody magnetic bead complex

The NP monoclonal antibody (NP mAb) used in this study was provided by MEDIAN Diagnostics. According to the manufacturer’s protocol for the Pierce Antibody Biotinylation Kit (Thermo Fisher Scientific), we labeled the NP mAb (1 mg/mL) with biotin. Then, 0.1 mg of biotin-labeled NP mAb was conjugated with 1 mg of Pierce streptavidin magnetic beads (Thermo Fisher Scientific).

### 2.5 RNP purification and concentration with NP mAb-magnetic bead complex (magnetic bead RNP purification and concentration method)

A total of 100 μL of the virus was mixed with 100 μL of RNP lysis buffer (50 mM Tris–HCl, pH 8.0; 100 mM KCl; 5 mM MgCl_2_; 1 mM DTT; 2% Triton X-100) (23). After mixing, 2 μL of NP mAb-magnetic bead complex was added, and the mixture was inverted several times. The beads were collected using a magnetic stand, and the supernatant was discarded. The beads were washed once with PBS. After washing, the beads were again collected on a magnetic stand, and elution was performed with 20 μL of 0.1 M glycine-HCl (pH 2.0). After elution, the beads were collected using a magnetic stand, and the supernatant was neutralized with 2 μL of 1 M Tris–HCl (pH 8.0) and used for the detection assay.

### 2.6 Real-time RT–PCR detection

Real-time RT–PCR was performed via the LiliF AIV H5 Real-time RT–PCR Kit (iNtRON) on a TaqMan qPCR system following the manufacturer’s protocol. To validate whether glycine-HCl buffer could elute RNPs from the NP-mAb-magnetic bead complex, we bound RNPs from the SNU50-5 strain to magnetic beads using the same method as previously described. We then used PBS and glycine-HCl as elution buffers. To compare the diagnostic efficacy of the conventional RNA extraction method (spin-column method) and the magnetic bead-based RNP purification and concentration method, we serially diluted the SNU50-5 virus from 10^6^ EID_50_/0.1 mL to 10^0^ EID_50_/0.1 mL, extracted RNA and purified RNPs from each dilution, and performed real-time RT–PCR to compare the Ct values.

### 2.7 Western blotting

To evaluate the effect of NP mAb-magnetic beads on NP protein capture and the effectiveness of glycine-HCl on eluting RNPs in a magnetic bead-based vRNP extraction method, RNPs from the SNU50-5 strain were purified using the magnetic bead-based method. One sample was eluted with glycine-HCl, while the other was eluted with PBS, followed by western blot analysis. Influenza A NP polyclonal antibody (Invitrogen, PA5-32242) was used as the primary antibody, and goat anti-rabbit IgG-HRP (BETHYL) was used as the secondary antibody.

### 2.8 Analytical sensitivity and specificity

To evaluate the analytical sensitivity of the PAM-independent Cas12a AIV detection method, RNA from 100 μL of A/Wild Duck/Korea/SNU50-5/2009 (H5N1) virus dilutions from 10^6^EID_50_/0.1 mL to 10^1^EID_50_/0.1 mL was extracted using the Patho Gene-spin DNA/RNA Extraction Kit (iNtRON) following the manufacturer’s instructions. PBS was used as the negative control. The extracted RNA was processed via the RT-RPA/PAM-independent Cas12a detection method, and fluorescence kinetics were measured with the Bio-Rad CFX Connect real-time PCR detection system. To evaluate the analytical specificity, all viruses were diluted to 10^6^ EID_50_/0.1 mL, except the infectious bronchitis virus, using the same detection protocol as in the sensitivity assessment. The diagnostic assay that combined the PAM-independent Cas12a detection method and RNP purification and concentration using magnetic beads was also evaluated for its analytic sensitivity and specificity. Except for magnetic bead RNP purification and concentration method, all the other procedures were as described above. The viruses used for specificity assessment are shown in Table 1.

### 2.9 Repeatability of magnetic bead RNP purification and PAM-independent Cas12a assay

To assess the repeatability of the magnetic bead RNP purification and PAM-independent Cas12a assay, the SNU50-5 virus was diluted to 10^3^ EID_50_/0.1 mL and subjected to the assay at different time points over one-day intervals.

## 3 Results

### 3.1 Design of primers for RT-RPA and crRNA for PAM-independent Cas12a-based detection

**Figure 1:**
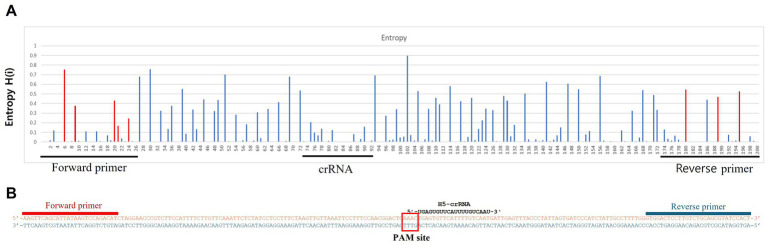
Entropy plot of the aligned avian influenza H5 gene sequences. To evaluate the sequence variability of the chosen conserved region (200 bp length), we trimmed only the selected region from the complete aligned sequences. We then performed positional nucleotide numerical summary (PNNS) and entropy calculations. Both the PNNS and entropy calculations were conducted using the website: http://entropy.szu.cz:8080/EntropyCalcWeb/. **(A)** Entropy plot for the H5 gene. The area marked in red indicates where we applied degenerate nucleotides or the primer set. **(B)** Sequence information for the primers and crRNA targeting the H5 gene. Notably, the H5 gene-targeting crRNA was designed without a canonical PAM sequence at its 5′ end.

To detect H5Nx viruses, we designed a primer/crRNA set targeting the H5 gene. We collected 8,504 complete H5 gene sequences of IAVs isolated from 1977 to 2020 from the Global Initiative on Sharing All Influenza Data (GISAID) database. The sequences were aligned via MAFFT, and the most highly conserved 200 bp region was selected (21). We chose three regions with the least variation to serve as forward and reverse primers and crRNA, and we performed PNNS and entropy calculations to quantify the nucleotide variability of the primers and crRNA regions (Figure 1) (22). According to the entropy plot, significant variation was observed at five distinct positions within the forward primer region and four distinct positions within the reverse primer region. Previous studies indicate that 2–5 mismatches in the primer binding region do not compromise the performance of the RPA assay (24, 25). Accordingly, the primers were designed to use only the minimal necessary degenerate bases and reduce the total number of primers, ensuring that each primer has no more than one mismatch with the target gene sequence. To address the observed sequence variability in the primer binding region, a set of primers was designed to accommodate nucleotide diversity at specific positions. In the forward primer set, degenerate bases were introduced at the five variable positions. However, incorporating degenerate bases at all five positions simultaneously would result in an excessive number of primer variants, potentially leading to false-positive reactions. To manage this, the forward primer set was divided into three (H5-RPA-F1, H5-RPA-F2, and H5-RPA-F3), each incorporating a subset of the degenerate positions. In the reverse primer set, high-frequency target gene sequences could be effectively covered without degenerate bases; therefore, it was designed with two primers (H5-RPA-R1 and H5-RPA-R2) without degenerate bases. The final primer set and crRNA sequences used are listed in Table 2. A comparative analysis was conducted to assess the mismatch sequences between our designed primers/crRNA and the collected gene sequences. The results demonstrated a high degree of congruity. Over 93% of the sequences presented one or no mismatch with both primers or crRNA (Table 3; Supplementary data). Even when the sequences of the recent clade 2.3.2.1c or clade 2.3.4.4b HPAIV H5Nx isolates (from 2020 to 2024) were compared with the selected primer/crRNA, approximately 92% of the isolates showed one or no mismatch with our primer and crRNA sequences (Supplementary Table S1; Supplementary data). This remarkable level of consistency underscores the robustness and reliability of the primers and crRNA that we designed for detection of the H5 genes of avian influenza viruses.

**Table 2:** Primer set, crRNA and reporter sequence used in this study.

Target		Primer set	PAM sequence	Sequence (5′–3′)	Primer length (bp)	Amplicon size
H5 gene	Forward primer	H5-RPA-F1		AGTGG**R**TA**Y**GCTGCAGACAAAGAATC	26	200 bp
		H5-RPA-F2		AGTGGTTA**Y**GCTGCAGACAAAGAATC		
		H5-RPA-F3		AGTGGATACGCTGCAGACA**RG**GA**G**TC		
	Reverse primer	H5-RPA-R1		AAGTTCAGCATTATAAGTCCAGACATCTA	29	
		H5-RPA-R2		AAGTTC**T**GCATT**G**TAAGTCCA**T**ACATCTA		
	crRNA	Not limited by PAM	AAAC	UGAGUGUUCAUUUUGUCAAU	20	
Reporter	Fluorescence			6-FAM/TTATT/BHQ/		
	LFD			6-FAM/TTATTATT/Bio/		

**Table 3:** Number of mismatches between the H5 gene of H5Nx isolates and the primer/crRNA set.

H5 gene			
Number of mismatches	Forward primer frequency	Reverse primer frequency	crRNA frequency
0	6140/8504 (72.20%)	5076/8504 (59.68%)	7071/8504 (83.14%)
1	2216/8504 (26.05%)	2896/8504 (34.05%)	1282/8504 (15.07%)
2	132/8504 (1.55%)	462/8504 (5.43%)	114/8504 (1.34%)
3	14/8504 (0.16%)	52/8504 (0.61%)	33/8504 (0.38%)
4	1/8504 (0.01%)	5/8504 (0.05%)	4/8504 (0.04%)
5	1/8504 (0.01%)	13/8504 (0.15%)	

### 3.2 Optimization of PAM-independent detection of the RT-RPA amplicon via Cas12a and H5-crRNA

**Figure 2:**
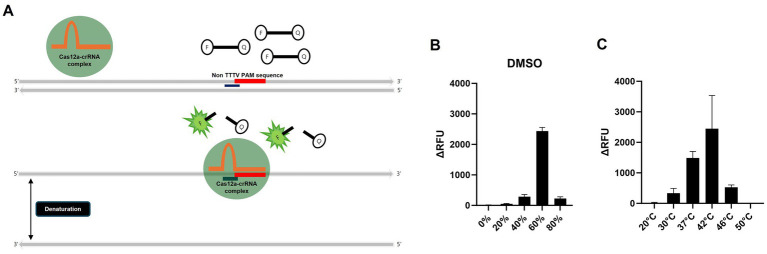
Schematic of the PAM-independent CRISPR/Cas12a assay and optimization of the PAM-independent CRISPR/Cas12a assay. **(A)** Schematic workflow of the PAM-independent CRISPR/Cas12 assay. When Cas12a targets double-stranded DNA (dsDNA), the binding might be inhibited if the PAM sequence is not TTTV. However, by using DMSO to denature the dsDNA into single-stranded DNA (ssDNA), the Cas12a-crRNA complex could potentially be activated without being limited by the PAM sequence. **(B)** Comparison of the detection efficiency of the PAM-independent Cas12a system in samples treated with 20, 40, 60, and 80% DMSO and those without DMSO treatment (0%). **(C)** Comparison of the detection efficiency of the RT-RPA and PAM-independent CRISPR/Cas12a assays under various reaction temperatures (20, 30, 37, 42, 46, and 50°C). Each data point is the mean ± standard deviation (SD) of triplicate experiments.

Dimethyl sulfoxide (DMSO) can denature double-stranded amplicons into single strands even at room temperature within 1 min, but if it does not severely inhibit crRNA binding and Cas12a activity, it may be useful for the PAM-independent detection strategy (26). To determine whether H5 gene can be detected independently of the PAM sequence, as outlined in the workflow in Figure 2A, we denatured the amplicon into ssDNA using DMSO, followed by a detection assay with Cas12a and H5-crRNA, which does not target the PAM sequence. We conducted RT-RPA using RNA extracted via the conventional spin–column method from a low-pathogenicity H5N1 AIV, 50–5_H5N1, and specific primers designed in this study (Tables 1, 2). To optimize the required DMSO concentration for detecting specific amplicons via Cas12a and H5-crRNA, we compared the effects of various concentrations of DMSO to the reaction mixture. Interestingly, among the various DMSO concentrations, only the RT-RPA amplicon containing 60% DMSO showed strong fluorescence after 20 min of incubation, due to the cleavage of the fluorescence reporter (Flu-Rep) in a PAM-independent manner, specifically activated Cas12a (Figure 2B). This result indicates that Cas12a can be activated to detect H5 genes without dependence on a specific PAM sequence. To further investigate the optimal temperature for the RT-RPA and PAM-independent Cas12a assays, both assays were performed under various reaction conditions ranging from 20 to 50°C. Detection efficiency was significantly reduced at temperatures other than 37 and 42°C, with 42°C identified as the most effective temperature for both RT-RPA amplification and Cas12a activation (Figure 2C).

### 3.3 Evaluation of the sensitivity and specificity of the PAM-independent detection of the RT-RPA amplicon via the Cas12a assay

**Figure 3:**
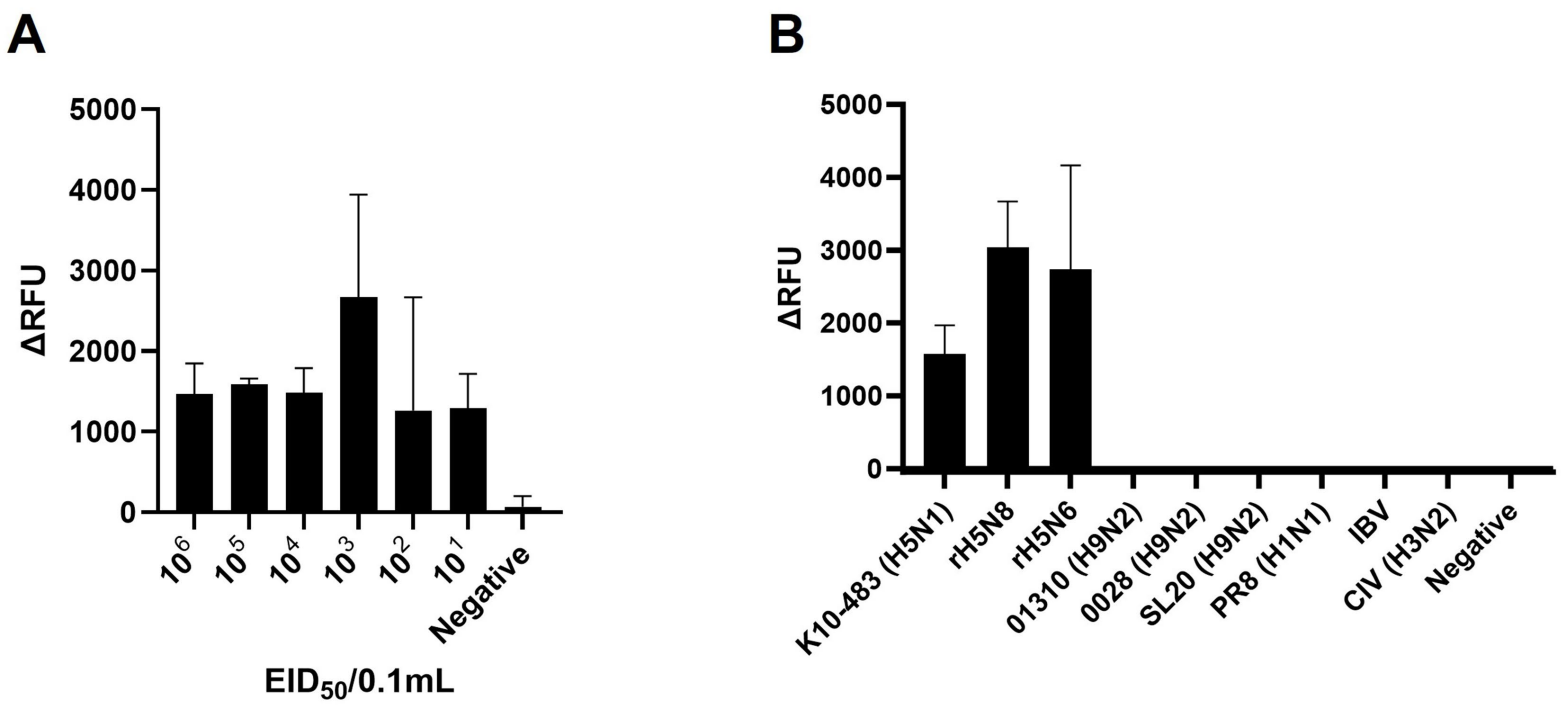
Sensitivity and specificity of PAM-independent Cas12a-based AIV detection. **(A)** To assess the detection sensitivity of the PAM-independent Cas12a-based method, the A/Wild Duck/Korea/SNU50-5/2009(H5N1) virus was subjected to 10-fold serial dilution from 10^6^ EID_50_/0.1 mL to 10^1^ EID_50_/0.1 mL in PBS. Afterwards, RNA was extracted by spin column method, and the RT-RPA/PAM-independent Cas12a assay was performed. **(B)** To evaluate the detection specificity of the RT-RPA/ PAM-independent Cas12a assay, viruses listed in Table 1 were diluted to a concentration of 10^6^ EID_50_/0.1 mL (with the exception of IBV, which was used undiluted). The procedure followed was identical to the one used for measuring sensitivity. Each data point is the mean ± standard deviation (SD) of triplicate experiments.

To evaluate the sensitivity, we performed RT-RPA and PAM-independent Cas12a assays using serial tenfold dilutions of 50–5_H5N1 RNA. When we used Flu-Rep and measured the fluorescence, the limit of detection (LoD) of our assay was 10^1^ EID_50_/0.1 mL within approximately 40 min (Figure 3A). We used only 2.5 μL from the approximately 50 μL elution of the extracted RNA, corresponding to less than 1 EID_50_ per reaction. Additionally, we evaluated the specificity of our assay using 8 IAVs, including 3 different pandemic clades of recombinant H5Nx strains (clade 2.3.2.1c rK10-483, clade 2.3.4.4a rH5N8, and clade 2.3.4.4c rH5N6), 5 different subtypes of IAV strains (01310_H9N2, 0028_H9N2, SL20_H9N2, PR8_H1N1, and CIV_H3N2) and an infectious bronchitis virus (IBV) (Table 1). No positive results were detected except for the H5Nx strains, demonstrating the specificity of the RT-RPA and PAM-independent Cas12a assay described herein (Figure 3B).

### 3.4 Optimization of anti-NP antibody-mediated magnetic bead RNP purification and concentration method and RNA preparation for the PAM-independent detection of the RT-RPA amplicon via the Cas12a assay

**Figure 4:**
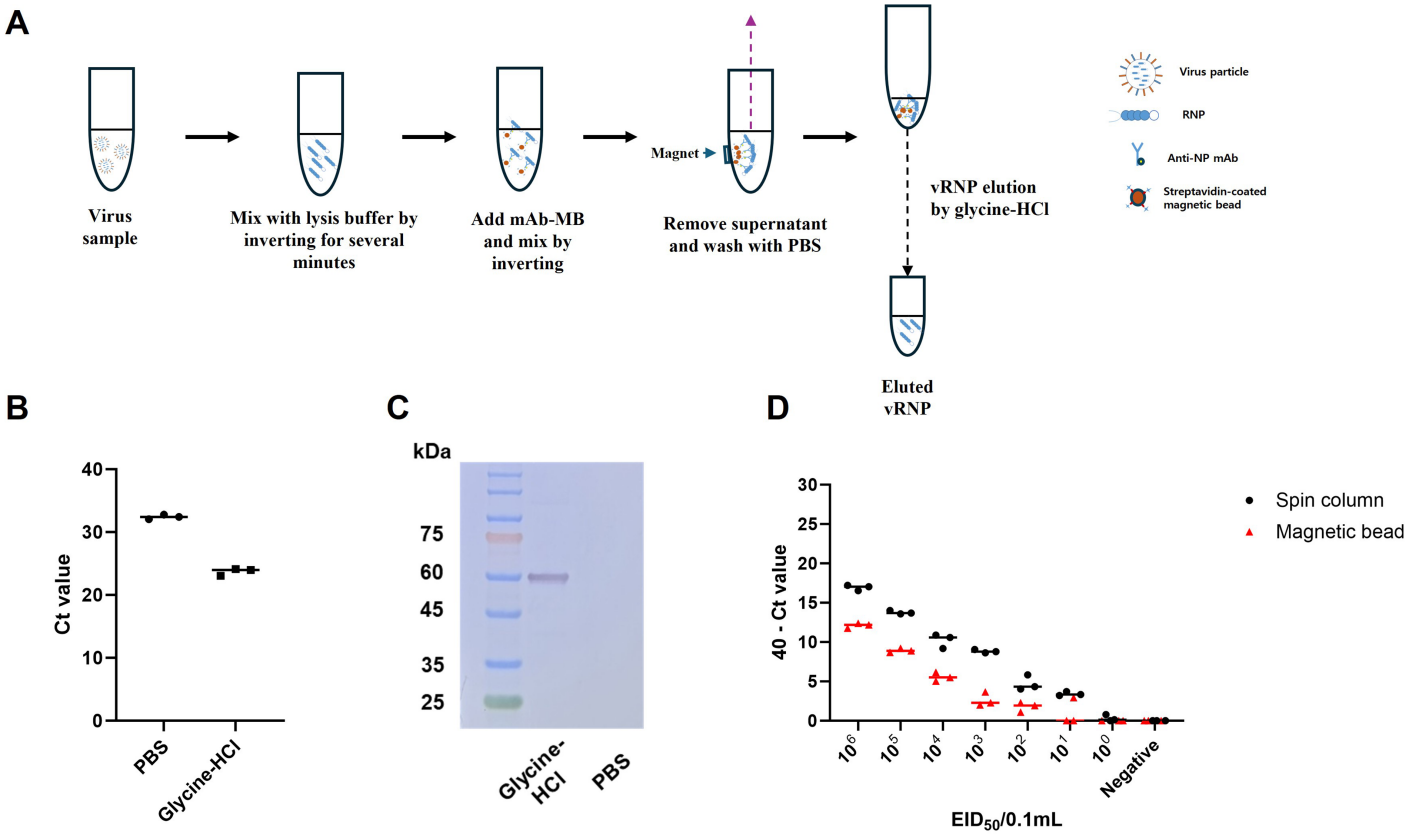
Schematic of the magnetic bead-based vRNP extraction method and comparison of qRT-PCR results for RNA extraction using spin columns and RNP purification and concentration using magnetic beads, including an assessment of the efficacy of the elution buffer. **(A)** Schematic representation of vRNP extraction using magnetic beads conjugated with NP mAb (mAb-MB). **(B)** To validate the effectiveness of glycine HCl for eluting RNPs, we performed qRT-PCR after eluting with each buffer using A/wild duck/korea/SNU50-5/2009 (H5N1) virus, which was diluted to 10^6^ EID_50_/0.1 mL. **(C)** Assessment of the effect of NP mAb-magnetic beads on RNPs capture and the impact of glycine-HCl on eluting RNPs in a magnetic bead-based vRNP extraction method using western blot analysis. **(D)** We compared the RNA extraction efficiency of the conventional spin column-based RNA extraction method with our newly developed magnetic bead-based RNP extraction method. After performing 10-fold serial dilutions of SNU50-5 virus from 10^6^ EID_50_/0.1 mL to 10^0^ EID_50_/0.1 mL in PBS, extraction using each method was followed by qRT-PCR. Each data point is the mean ± standard deviation (SD) of triplicate experiments.

Previously, we suggested that anti-NP antibodies could be useful for purifying viral RNA in the form of RNPs, achieving a purification level comparable to conventional RNA extraction methods and purified RNP with magnetic bead can be directly added to real time RT-PCR reaction without any adverse effect (27). However, the purified RNP bound to magnetic beads were not appropriate for direct application in RT-RPA, which is conducted at a relatively low isothermal reaction temperature of 42°C. Therefore, we tried to find a buffer to dissociate both RNPs from magnetic beads and RNA from NPs without adverse effects on the RT-RPA and PAM-independent Cas12a assays (Figure 4A). Finally, we used acidic glycine HCl buffer (pH 2.0) to dissociate RNA from the NPs following neutralization with 1 M Tris–HCl (pH 8.0). We evaluated the effect of NP mAb-magnetic beads on RNPs capture and verified the use of glycine HCl buffer in comparison with that of PBS via qRT–PCR and western blot analysis. The glycine-HCl buffer displayed superior performance to PBS in the preparation of RNA samples, as demonstrated by a Ct value difference of more than 8.5 in the qRT–PCR results (Figure 4B). Additionally, western blot analysis revealed NP-specific bands only when glycine HCl buffer was used (Figure 4C). These findings demonstrate that NP mAb-magnetic beads effectively capture both RNA and NP protein (RNPs) and that glycine HCl buffer enables efficient elution of RNPs from the anti-NP antibody-mediated magnetic beads. We also compared the efficiency of our magnetic bead-based anti-NP antibody-mediated RNP purification and concentration method with that of the conventional spin column-based RNA extraction method. While the spin column extraction method exhibited 10-fold greater sensitivity, the magnetic bead RNP purification and concentration method also displayed a remarkably high level of sensitivity, enough to show the same positive result for 10^2^ EID_50_/0.1 mL and slightly weaker positive results for 10^1^ EID_50_/0.1 mL. Moreover, the spin column method yielded 3/3 positive results, while our method yielded 1/3 positive results (Figure 4D).

### 3.5 Integration of the anti-NP antibody-mediated magnetic bead RNP purification and concentration method with an RT-RPA/PAM-independent Cas12a assay for specific H5Nx AIV detection

**Figure 5:**
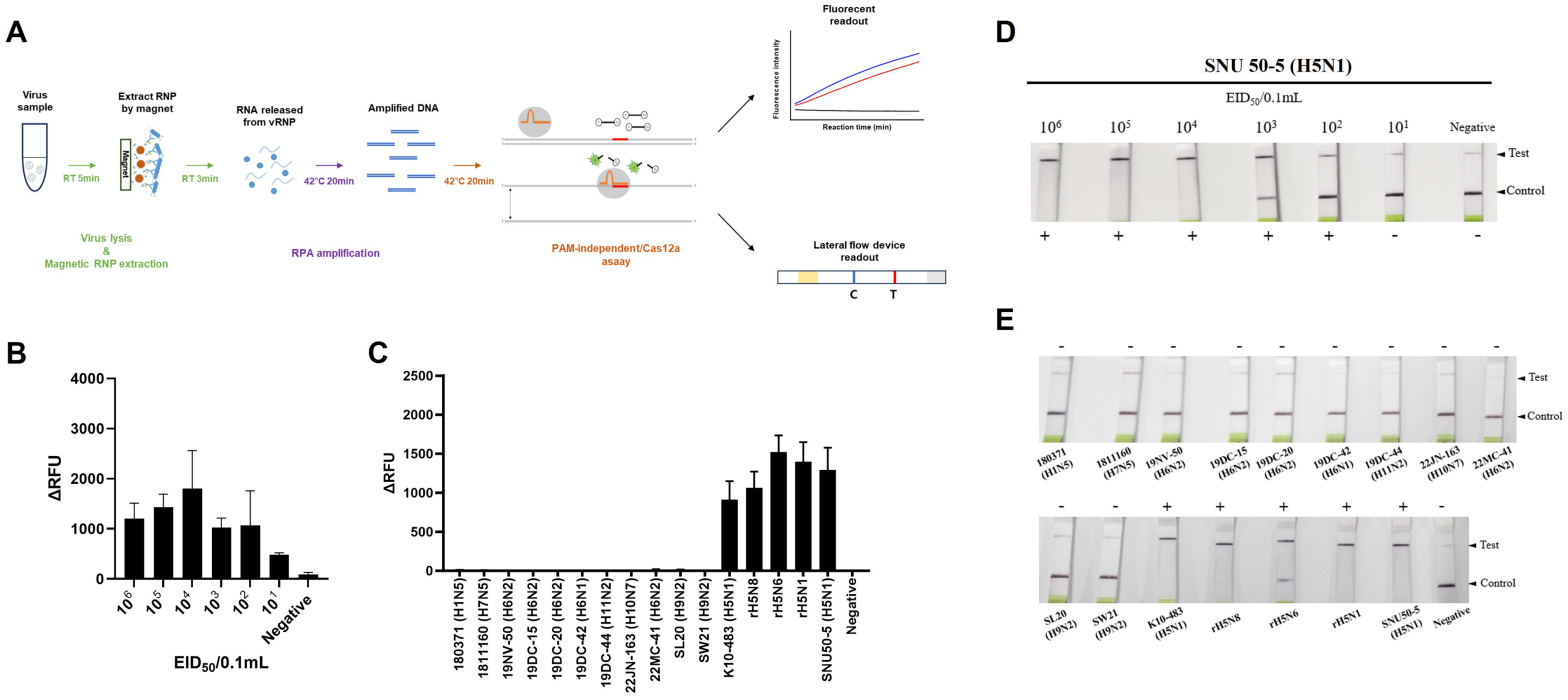
Sensitivity and specificity of magnetic bead RNP purification/PAM-independent Cas12a-based AIV detection. **(A)** Schematic of the magnetic bead-based RNP purification method with the PAM-independent/CRISPR-Cas12a assay. **(B,D)** Fluorescence and lateral flow strip (LFS) detection sensitivity for the SNU50-5 virus using magnetic bead RNP purification combined with the RT-RPA/PAM-independent Cas12a assay. The virus was subjected to tenfold serial dilution from 10^6^ EID_50_/0.1 mL to 10^1^ EID_50_/0.1 mL in PBS. Afterwards, RNP was purified and concentrated by mAb-MB, and the RT-RPA/PAM-independent Cas12a assay was performed. **(C,E)** Fluorescence and LFS detection specificity for the various subtypes of avian influenza virus (Table 1). Each data point is the mean ± standard deviation (SD) of triplicate experiments.

We combined an anti-NP antibody-mediated RNA preparation method with an RT-RPA/PAM-independent Cas12a assay to evaluate the performance of these methods for the detection of specific H5Nx AIVs (Figure 5A). First, we purified and concentrated RNA from 10-fold serial dilutions of the 50–5_H5N1 strain (from 10^6^ EID_50_/0.1 mL to 10^1^ EID_50_/0.1 mL) via the anti-NP antibody-mediated RNA preparation method and performed an RT-RPA/PAM-independent Cas12a assay to test the sensitivity. The LoDs of the fluorescence device and the LFS were determined to be 10^1^ EID_50_/0.1 mL and 10^2^ EID_50_/0.1 mL, respectively (Figures 5B,C). To evaluate the specificity of the integrated method, we tested various subtypes of AIV strains (Table 1). Only the H5Nx AIV strains yielded positive results in both the fluorescence and LFS detection methods (Figures 5D,E). The repeatability of the combined detection assay was also evaluated at different time points over one-day intervals. Our combined detection assay demonstrated consistent results over a 3-day period, confirming its high repeatability (Supplementary Figure S1). To evaluate the reliability of the magnetic bead RNP purification and PAM-independent Cas12a assay, we analyzed its performance using clinical samples. A total of 29 clinical samples were used, including 15 confirmed RT-qPCR-positive samples and 14 confirmed RT-qPCR-negative samples. The magnetic bead RNP purification and PAM-independent Cas12a assay detected 12 H5-RT-qPCR-positive samples and 14 H5-RT-qPCR-negative samples, demonstrating a diagnostic sensitivity of 80% and a diagnostic specificity of 100% (Table 4; Supplementary Figure S2).

**Table 4:** Performance of magnetic bead RNP purification/PAM-independent Cas12a-based AIV detection in clinical sample.

		RT-qPCR		
		Positive	Negative	Total
RNP purification/PAM-independent Cas12a-based AIV detection	Positive	12	0	12
	Negative	3	14	17
	Total	15	14	29
Sensitivity	80%			
Specificity	100%			

## 4 Discussion

Among HPAIVs, the H5 subtype virus has caused major global outbreaks in wild birds and poultry in Asia, Europe, Africa, and North America since 2014 (2). Since the COVID-19 pandemic, numerous point-of-care diagnostic methods for viruses have been developed, overcoming the limitations of traditional diagnostic approaches. Several groups have reported COVID-19 diagnostic assays that use RT-RPA or RT-LAMP, with or without CRISPR-mediated target detection (13, 14, 16, 28, 29), but there are few reports of their application to H5Nx influenza viruses. Additionally, the RT-LAMP method poses challenges for diagnosing highly variable H5 genes because of the need to design multiple primers. While the RT-RPA method uses only two primers, it has the drawback of producing a high number of false-positive results when used alone (30, 31).

Our study provides a simple, fast, centrifuge-free RNA isolation method based on anti-NP antibody-magnetic bead complex beads combined with an RT-RPA/PAM-independent Cas12a assay for the detection of H5Nx AIVs. Our study highlights the efficacy and reliability of the designed primer/crRNA set for detecting universal H5Nx AIVs. Typically, the RPA assay uses longer primers and requires lower incubation temperatures than PCR and other isothermal amplification methods do. This allows RPA to be more tolerant of genetic variations in primer binding sites. Previous studies have shown that the performance of the RPA assay is not affected by 2–5 mismatches in the primer binding region (24, 25). Therefore, the primers were designed to minimize the introduction of degeneracy and the number of primers while ensuring that there was no more than one mismatch between the primer and the target DNA sequence. Furthermore, the upstream PAM sequence in the target DNA is required for Cas12a activation to initiate trans-ssDNA cleavage. However, when Cas12a and crRNA target ssDNA, trans-cleavage can be activated even with mutations in the PAM sequence, and the trans-cleavage activity is minimally affected even with a single mismatch in the target strand (13, 32). The crRNA targeting the H5 gene was designed without the conventional PAM sequence, 5’-TTTV-3′, and, similar to the primer design, ensuring that there was no more than one mismatch with the target strand. Consequently, our comparative analysis demonstrated a high degree of congruity between the designed primers/crRNA and the collected H5 gene sequences, with over 93% of the sequences showing one or no mismatch (Table 3). Notably, even when recent H5Nx isolates from clades 2.3.2.1c and 2.3.4.4b (from 2020 to 2024) were analyzed, approximately 92% of the isolates presented one or no mismatch with our primer and crRNA sequences (Supplementary Table S2). In addition, we performed specificity tests of our newly developed assay using 5 different H5Nx viruses and 15 non-H5Nx subtype viruses (Figure 5E). Among the viruses used in the specificity test, the rH5N6 virus had a total of 3 mismatches: 1 with the forward primer, 1 with the reverse primer, and 1 with the crRNA. The rH5N1 virus had a total of 4 mismatches: 2 with the forward primer and 2 with the reverse primer. Despite these mismatches, both viruses were successfully detected. In contrast, non-H5Nx subtype viruses had at least 12 mismatches, and none of these viruses were detected (Table 1; Supplementary Table S3). Overall, the primer and crRNA set designed in our study exhibited high specificity and reliability, accurately detecting H5Nx viruses despite minor mismatches and excluding non-H5Nx subtypes. Additionally, they maintained high match accuracy with both recent and historical isolates, demonstrating robustness against genetic variations. However, updates are required to ensure the detection of newly emerging variants.

We also addressed several limitations of current CRISPR/Cas diagnostic methods by developing a CRISPR/Cas12a detection system that is independent of the PAM sequence. The Cas13 enzyme, despite not requiring a PAM sequence, needs a longer spacer and an additional transcription step, complicating its use for the variable H5 gene. The Cas12 enzyme, while simpler because of its shorter spacer length and ability of direct DNA targeting, requires a specific PAM sequence (5’-TTTV-3′), which complicates crRNA design for H5 detection. By using single-stranded DNA (ssDNA) instead of double-stranded DNA (dsDNA) as the crRNA target, we successfully activated trans-cleavage activity regardless of the PAM sequence. This was most effectively achieved with 60% DMSO, which aligns with previous studies indicating that 60% DMSO is optimal for denaturing dsDNA into ssDNA (Figure 2B) (26). The RT-RPA and PAM-independent Cas12a assay demonstrated optimal detection efficiency at 42°C, while exhibiting a marked decline in detection efficiency under conditions outside the temperature range of 37–42°C, indicating a strong temperature dependency (Figure 2C). The reverse transcriptase used in RT-RPA achieves its optimal activity at 42°C, and RPA reaction operates efficiently within the range of 37–42°C (11). Additionally, the Cas12a enzyme utilized in this study requires a minimum activation temperature of at least 28°C (33). Therefore, the narrow range of optimal reaction temperatures appears to be due to the temperature dependency of the reverse transcriptase and RPA reactions. Based on these findings, the final reaction conditions were set to a DMSO concentration of 60% and a reaction temperature of 42°C. Additionally, our RT-RPA/PAM-independent Cas12a assay, which uses 60% DMSO and a reaction temperature of 42°C, exhibited a detection sensitivity as low as 10^1^ EID_50_/0.1 mL for the virus. Designing crRNA without the need to consider the PAM sequence offers significant advantages. It allows greater flexibility and ease in the design process, as researchers are not constrained by the need to incorporate a specific PAM sequence. This is especially advantageous when addressing genes with high variability, such as the hemagglutinin gene, which might have sequence variations across different strains or undergo changes over time.

In addition to the PAM-independent CRISPR–Cas12a assay, the magnetic bead RNP purification and concentration method offers an efficient, specific, and rapid alternative to conventional spin column-based RNA extraction methods. Because RNA is bound to the surface of RNPs (19, 23), RNA can be coextracted during RNP isolation from virus samples via magnetic beads conjugated with anti-NP monoclonal antibodies. In our previous study, we confirmed that anti-NP monoclonal antibodies and magnetic beads could serve as an alternative to the conventional spin column RNA extraction method (27). However, a limitation was that antibodies and beads had to be added separately for each experiment, making the process complex and lengthening the reaction time. Furthermore, while PCR can directly apply the RNP-bead complex by denaturing it at high temperatures, this approach is unsuitable for RT-RPA due to its lower reaction temperature (42°C). Therefore, in this study, we simplified the process by using NP mAb-magnetic bead, binding biotinylated anti-NP monoclonal antibodies with streptavidin-coated magnetic beads. Additionally, by eluting RNA from RNPs bound to magnetic beads using a glycine-HCl buffer, we enabled compatibility with RT-RPA. This significantly streamlined the process, reducing the reaction time while maintaining high extraction efficiency.

Our magnetic bead RNP purification and concentration method, combined with the PAM-independent CRISPR-Cas12a assay, achieved a detection limit of 10^1^ EID_50_/0.1 mL via fluorescence detection and 10^2^ EID_50_/0.1 mL via LFS-mediated detection (Figures 5B,D). Furthermore, only the H5Nx AIV strains displayed positive outcomes in both the fluorescence and LFS-mediated detection, showing 100% specificity (Figures 5D,E). According to previous reports, the viral titers determined from oropharyngeal and cloacal swabs of chickens experimentally infected with avian influenza virus range from 10^2^ to 10^4^ EID_50_/0.1 mL (34–36), demonstrating that our method has sufficiently high sensitivity for detecting viruses in clinical samples. When diagnosing clinical samples, specificity is crucial, in addition to sensitivity. Fecal and swab samples often contain various biological substances and contaminants that can hinder accurate viral gene detection, leading to nonspecific amplification during PCR processes (37). To mitigate these issues, a detection method with high specificity is essential. Unlike the spin column method or other magnetic bead-based extraction techniques (38–40), our method employs virus-specific anti-NP monoclonal antibodies for RNP purification and concentration, increasing detection specificity. Furthermore, by utilizing a CRISPR-Cas12a assay with virus-specific crRNA following RPA amplification, we anticipate achieving increased specificity in genetic detection. This dual approach ensures robust and precise viral diagnosis, significantly reducing the likelihood of nonspecific reactions. Our magnetic bead RNP purification and PAM-independent Cas12a assays demonstrated 100% diagnostic specificity with clinical samples, as expected (Table 4; Supplementary Figure S2). This result confirms that various biological substances present in clinical samples did not interfere with the specificity of our assay. However, the diagnostic sensitivity was 80%, with three out of 15 positive samples not being detected. Although not explicitly shown in the data, the false-negative clinical samples had Ct values exceeding 37, suggesting that these samples were beyond the detection limit of our assay. These findings highlight the need to further optimize the assay’s sensitivity to reliably detect samples with low viral loads while maintaining its high specificity. The cost analysis reveals that the traditional RNA extraction and qPCR method involves an expense of $8.12 per reaction, whereas the RNP purification/PAM-independent Cas12a-based AIV detection method costs only $2.9 per reaction. This indicates that the our detection method is approximately 2.8 times more cost-effective than the conventional approach. These findings underscore the economic advantage of our detection method, particularly when compared to traditional diagnostic techniques (Supplementary Table S4). However, several limitations need to be addressed. We did not address the long-term stability of the reagents used in our assay in their mixed state. While the individual reagents used for magnetic bead RNP purification, RT-RPA and PAM-independent Cas12a detection are stable for at least 1 year under recommended storage conditions, the stability of the reagents in their combined form remains unknown. For real-world applications, further studies are required to assess the long-term stability of the reagents when mixed. Moreover, it is important to verify whether different combinations of monoclonal antibodies affect diagnostic performance. Additionally, our current protocol requires a fixed temperature of 42°C for isothermal amplification, introducing a temperature dependency. Future studies should investigate whether the assay can function effectively at lower temperatures or even at room temperature to broaden its applicability.

Taken together, our findings offer an effective and rapid diagnostic method for the detection of H5Nx viruses, which are a global threat to wild birds and poultry. By combining a magnetic bead RNP purification and concentration method with an RT-RPA/PAM-independent Cas12a assay detection system, we were able to circumvent the traditional complexities and time-consuming steps associated with HPAIV surveillance. This novel approach is promising not only for the potential application of this method in H5Nx virus detection but also for providing a general template that could be adapted for the detection of other rapidly mutating RNA viruses.

## Data Availability

The datasets presented in this study can be found in online repositories. The names of the repository/repositories and accession number(s) can be found in the article/Supplementary material.

## Footnotes

^1^ https://platform.epicov.org/epi3/frontend#5c38a7

^2^ http://entropy.szu.cz:8080/EntropyCalcWeb/
