# Supplementary material for: Rapid and specific on-site H5Nx avian influenza diagnosis via RPA and PAM-independent CRISPR-Cas12a assay combined with anti-NP antibody-based viral RNA purification
Source: Front Vet Sci. 2025 Jan 17;12:1520349. doi: 10.3389/fvets.2025.1520349 (PMC11782159; doi:10.3389/fvets.2025.1520349)
Supplement: Supplementary file 1 [file Data_Sheet_1.docx]

Supplementary Tables & Figures

# Supplementary Tables

| Strain name | Subtype | Abbreviation | Accession number or reference |
| --- | --- | --- | --- |
| A/Anser fabalis/South Korea/180371/2018 | H1N5 | 180371 | PQ533814 |
| A/Anas platyrhynchos/South Korea/1811160/2018 | H7N5 | 1811160 | PQ533815 |
| A/Anser fabalis/South Korea/19NV-50/2019 | H6N2 | 19NV-50 | OQ296920 |
| A/Anser fabalis/South Korea/19DC-15/2019 | H6N2 | 19DC-15 | PQ533832 |
| A/Anser fabalis/South Korea/19DC-20/2019 | H6N2 | 19DC-20 | PQ533833.1 |
| A/Anser brachyrhynchus/South Korea/19DC-42/2019 | H6N1 | 19DC-42 | OQ296896 |
| A/Anser brachyrhynchus/South Korea/19DC-44/2019 | H11N2 | 19DC-44 | OQ296904 |
| A/Anser albifrons/South Korea/22JN-163/2022 | H10N7 | 22JN-163 | OQ296832 |
| A/Anser albifrons/South Korea/22MC-41/2022 | H6N2 | 22MC-41 | OQ296848. |
| A/chicken/Korea/SL20/2020 | H9N2 | SL20 | OP247632 |
| A/Greylag Goose/South Korea/SW21/2021 | H9N2 | SW21 | OP268457 |
| A/Mandarin duck/Korea/K10-483/2010,2.3.2.1 c rH5N1(p) * | H5N1 | K10-483 | JF699677 |
| 2.3.4.4a rH5N8(p) *^a^ | H5N8 | rH5N8 | PQ554514, [1] |
| 2.3.4.4c rH5N6(p) *^b^ | H5N6 | rH5N6 | PQ554515, [2] |
| 2.3.4.4b rH5N1(p) *^c^ | H5N1 | rH5N1 | PQ554516 |
| A/wild duck/korea/SNU50-5/2009 | H5N1 | SNU50-5 | JX497768 |
| A/chicken/Korea/01310/2001 | H9N2 | 01310 | EU253561 |
| A/Korea/KBNP-0028/2000 | H9N2 | 0028 | EF620900 |
| A/Puerto Rico/8/34 | H1N1 | PR8 | EPI29937 |
| A/canine/Korea/SH6/2017 (CIV) | H3N2 | CIV | EPI2468368 |
| Korea/SNU19018/19, Infectious bronchitis (IB) |  | IBV | OP765956 |

**Supplementary Table 1.** List of viruses used for this study

* The 2+6 recombinant viruses generated through reverse genetics. The HA and NA gene segments were derived from those listed in the table, while the remaining six internal gene segments were derived from the PR8 strain

^a^ Recombinant H5N8 influenza virus generated using consensus HA and NA sequences from clade 2.3.4.4a H5N8 HPAIVs isolated in Asia between 2014 and 2016, with the multi-basic cleavage site in the HA gene replaced by an ASGR-coding sequence to attenutae virulence.

^b^ Recombinant H5N6 influenza virus generated using most frequent HA and NA sequences from clade 2.3.4.4c H5N6 HPAIVs isolated between 2014 and 2016, with the multi-basic cleavage site in the HA gene replaced by an ASGR-coding sequence to attenutae virulence.

^c^ Recombinant H5N1 influenza virus generated using consensus HA and NA sequences from clade 2.3.4.4b H5N1 HPAIVs isolated in Asia between 2021 and 2023, with the multi-basic cleavage site in the HA gene replaced by an ASGR-coding sequence to attenutae virulence.

**(A)**

| H5 gene (2.3.2.1c, 2020.01.01-2024.03.01) | | | |
| --- | --- | --- | --- |
| Number of mismatches | Forward primer frequency | Reverse primer frequency | crRNA frequency |
| 0 | 174/181 (96.13%) | 135/181 (74.58%) | 27/181 (14.91%) |
| 1 | 7/181 (3.86%) | 37/181 (20.44%) | 154/181 (85.08%) |
| 2 |  | 9/181 (4.97%) |  |

**(B)**

| H5 gene (2.3.4.4b, 2020.01.01-2024.03.01) | | | |
| --- | --- | --- | --- |
| Number of mismatches | Forward primer frequency | Reverse primer frequency | crRNA frequency |
| 0 | 4406/11848 (37.18%) | 12/11848 (0.1%) | 11619/11848 (98.06%) |
| 1 | 7277/11848 (61.41%) | 10924/11848 (92.2%) | 228/11848 (1.92%) |
| 2 | 163/11848 (1.37%) | 937/11848 (7.9%) | 1/11848 |
| 3 | 2/11848 (0.01%) | 5/11848 (0.04%) |  |

**Supplementary Table 2.** Number of mismatches between recent HPAIV H5Nx isolates and the H5 gene primer/crRNA set

| **Virus** | **Forward primer (5’ – 3’)** | **Mismatch** | **Reverse primer (3’ – 5’)** | **Mismatch** | **crRNA (5’ – 3’)** | **Mismatch** | Total  mismatch |
| --- | --- | --- | --- | --- | --- | --- | --- |
| 180371 | **TCC** GGT TAC GC**A** GC**T** GA**T** **C**A**G** **A**A**G** **AG** | 12 | T**G**G ATG T**T**T GGA C**A**T ACA ATG CAG AA**T** T**G** | 5 | ATT GA**G** AAA ATG AAC ACT CA | 1 | 18 |
| 1811160 | **GAG** GGA **ACA** GCT GCA GA**T** **T**A**C** **A**AA **AG** | 12 | **C**AG A**G**G T**G**T GG**T** C**C**T ATA ATG CTG AAC T**A** | 6 | AT**A** GA**A** AAA A**CC** AAC **CAA** CA | 7 | 25 |
| 19NV-50 | **TCA** GGA TAT GC**A** GCA GAC AAA GA**G** **AG** | 7 | TAG ATG TCT GGA C**A**T A**C**A ATG CTG AAC T**G** | 3 | ATT GA**T** AAA ATG AAC AC**A** CA | 2 | 12 |
| 19DC 15 | **TCA** GGA TAT GC**A** GCA GAC AAA GA**G** **AG** | 7 | TAG ATG T**T**T GGA C**A**T A**C**A ATG CTG AAC T**G** | 4 | ATT GA**T** AAA ATG AAC AC**A** CA | 2 | 13 |
| 19DC-20 | **TCA** GGA TAT GC**A** GCA GAC AAA GA**G** **AG** | 7 | TAG ATG T**T**T GGA C**A**T A**C**A ATG CTG AAC T**G** | 4 | ATT GA**T** AAA ATG AAC AC**A** CA | 2 | 13 |
| 19DC-42 | **TCA** GGA TAT GC**A** GCA GAC AAA GA**G AG** | 7 | TAG ATG T**T**T GGA C**A**T A**C**A ATG CTG AAC T**G** | 4 | ATT GA**T** AAA ATG AAC AC**A** CA | 2 | 13 |
| 19DC-44 | A**CA** GGT **ATA** GC**A** GCA GAC AA**G** GAA TC | 7 | T**T**G A**CA** T**C**T GG**T** C**G**T ACA ATG CA**C** AAC T**C** | 8 | **G**T**C** GAC A**G**A ATG AA**T** AC**A** CA | 5 | 20 |
| 22JN-163 | A**C**T GGT **C**A**G** GCT GC**G** GA**T** **T**A**C** **A**A**G AG** | 11 | **C**AG A**CA** T**C**T GGA C**G**T AT**C** A**A**G CTG AA**T** T**G** | 8 | AT**A** GA**A** AA**G** A**CA** AAC AC**A** **G**A | 7 | 26 |
| 22MC-41 | **TCA** GGA TAT GC**A** GCA GAC AAA GA**G AG** | 7 | TAG ATG T**T**T GGA CAT ACA ATG C**T**G AAC T**G** | 4 | ATT GA**T** AA**G** ATG AAC AC**A** CA | 3 | 14 |
| SL20 | **GT**T GGT **ATG** GC**A** GCA GA**T** A**G**A GA**T** TC | 9 | **A**AG AT**A** TAT GG**G** C**A**T A**T**A ATG CAG AA**T** T**G** | 7 | **G**T**C** GAC AAA ATG AAC A**AG** CA | 4 | 20 |
| SW21 | A**C**T GGA **ATG** GCT GCA GA**T** A**G**A GAA TC | 6 | **A**AG AT**A** T**T**T GG**G** CTT ACA ATG CAG AA**T** T**G** | 6 | AT**A** GAC AAA ATG AAC A**AA** CA | 3 | 15 |
| K10-483 | AGT GGG TAC GCT GCA GAC AAA GAA TC | 0 | TAG ATG T**T**T GGA CTT ATA ATG CTG AAC TT | 1 | ATT GAC AAA ATG AAC ACT CA | 0 | 1 |
| rH5N8 | AGT GGG TAC GCT GCA GAC AAA GAA TC | 0 | TAG ATG TCT GGA CTT ATA ATG CTG AAC TT | 0 | ATT GAC AAA ATG AAC ACT CA | 0 | 0 |
| rH5N6 | AGT GGG TAC GCT GCA GAC A**G**A GAA TC | 1 | TAG ATG TCT GGA CTT A**C**A ATG CTG AAC TT | 1 | AT**C** GAC AAA ATG AAC ACT CA | 1 | 3 |
| rH5N1 | AGT GGG TAC GCT GCA GA**T** AA**G** GAA TC | 2 | TAG ATG T**G**T GGA C**C**T ATA ATG CTG AAC TT | 2 | ATT GAC AAA ATG AAC ACT CA | 0 | 4 |
| SNU50-5 | AGT GGA TAC GCT GCA GAC AAG GAG TC | 0 | TAG ATG TCT GGA CTT ATA ATG CTG AAC TT | 0 | ATT GAC AAA ATG AAC ACT CA | 0 | 0 |
| 01310 | **GT**T GGA **ATA** GC**C** GCA GAC AAA GAA TC | 6 | **AG**G A**CA** T**C**T GG**G** C**G**T ACA ATG C**A**G AA**T** T**A** | 10 | AT**C** GAC AAA ATG AAC A**AG** CA | 3 | 19 |
| 0028 | **GT**T GGA **ATG** GC**C** GCA GAC AA**G** GAA TC | 7 | **AG**G A**CA** T**C**T GG**G** C**G**T ACA ATG C**A**G AA**T** T**A** | 10 | AT**C** GAC AAA ATG AAC A**AG** CA | 3 | 20 |
| PR8 | **TCA** GG**C** TAT GC**A** GC**G** GA**T** **C**AA AAA **AG** | 10 | **TG**G A**CA** T**T**T GGA C**A**T ATA ATG C**A**G AA**T** T**G** | 9 | AT**C** GA**G** AAA ATG AAC A**T**T CA | 3 | 22 |
| CIV | A**CA** GGA **C**A**A** GC**A** GCA GAC **CTT** AAA **AG** | 10 | TAG AT**C** T**T**T GG**T** CTT ACA ATG C**C**G A**G**C TT | 5 | ATT GA**A** AAA A**C**G AA**T** **GAA** **A**A | 7 | 22 |

**Supplementary Table 3.** Number of mismatches and the mismatch sequences between the AIVs used in the specificity test and the primers/crRNA set.

Sequence highlighted in bold red indicate mismatches with the selected primers/crRNA set

**(A)**

| **Traditional RNA extraction and qPCR methods** | | | | |
| --- | --- | --- | --- | --- |
| **Component** | **Amount** | **Reaction** | **Cost ($)** | **Cost/1 reaction ($)** |
| qRT-PCR kit | 1 kit (MEDIAN DIAGNOSTIC) | 96 | 431.54 | 4.4952 |
| RNA extraction kit | 1 kit (iNTRON) | 50 | 181.64 | 3.6328 |
|  |  |  | Total | 8.128 |

**(B)**

| **RNP purification/PAM-independent Cas12a-based AIV detection** | | | | |
| --- | --- | --- | --- | --- |
| **Component** | **Amount** | **Reaction** | **Cost ($)** | **Cost/1 reaction ($)** |
| Pierce™ Streptavidin Magnetic Beads | 1 vial (Pierce, 88816) | 2500 | 1020 | 0.4080 |
| Pierce™ Antibody Biotinylation Kit for IP | 1 kit (Pierce, 90407) | 800 | 339.09 | 0.4239 |
| RevertAid Reverse Transcriptase | 1 vial (Thermo Scientific, EP0441) | 150 | 62.35 | 0.4156 |
| TwistAmp® Basic | 1 kit (TwistDx, TABAS03KIT) | 480 | 464.7 | 0.9681 |
| EnGen® Lba Cas12a (Cpf1) | 1 vial (NEB, M0653S) | 140 | 81.92 | 0.5851 |
| Reporter | 1 vial | 2000 | 142.49 | 0.0712 |
| Primer | Forward primer | 2000 | 8.91 | 0.0045 |
|  | Reverse primer |  | 12.91 | 0.0065 |
| crRNA | 1 vial (IDT Korea) | 4000 | 85.63 | 0.0214 |
|  |  |  | Total | 2.9043 |

**Supplementary Table 4.** Cost analysis between traditional RNA extraction and qPCR method and RNP purification/PAM-independent Cas12a-based AIV detection method

# Supplementary Figure

**Supplementary Figure 1. Repeatability of magnetic bead RNP purification and PAM-independent Cas12a assay.** The SNU50-5 virus, diluted to 10^3^ EID_50_/0.1mL, was subjected to magnetic bead RNP purification and PAM-independent Cas12a assay at different time point over one-day intervals. Each data point is the mean ± standard deviation (SD) of triplicate experiments


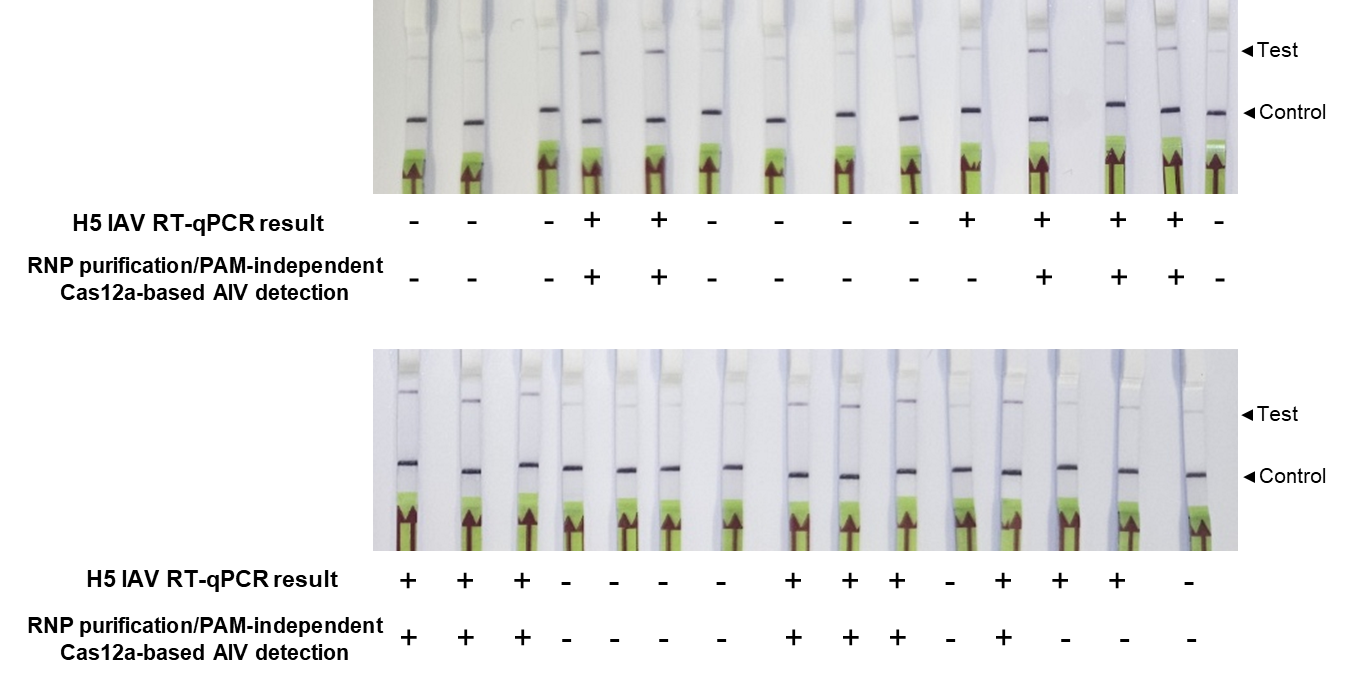


**Supplementary Figure 2. Magnetic bead RNP purification and PAM-independent Cas12a assay detection performance for clinical samples using lateral flow strip (LFS).** We performed magnetic bead RNP purification and PAM-independent Cas12a assays on a total of 29 wild bird fecal samples, including 15 RT-qPCR-positive and 14 RT-qPCR-negative samples, and evaluated detection efficiency using lateral flow strips. '+' indicates a positive result, and '-' indicates a negative result.

1. An, S.-H., et al., *Bioengineering a highly productive vaccine strain in embryonated chicken eggs and mammals from a non-pathogenic clade 2· 3· 4· 4 H5N8 strain.* Vaccine, 2019. **37**(42): p. 6154-6161.

2. An, S.-H., et al., *Improvement of PR8-derived recombinant clade 2.3. 4.4 c H5N6 vaccine strains by optimization of internal genes and H103Y mutation of hemagglutinin.* Vaccines, 2020. **8**(4): p. 781.
